# Influence of Extrusion Cooking Parameters on Antioxidant Activity and Physical Properties of Potato-Based Snack Pellets Enriched with Cricket Powder

**DOI:** 10.3390/molecules30234612

**Published:** 2025-12-01

**Authors:** Maciej Combrzyński, Jakub Soja, Michał Staniak, Beata Biernacka, Karolina Wojtunik-Kulesza, Marek Gancarz, Tomasz Oniszczuk, Magdalena Kręcisz, Jarosław Szponar, Anna Oniszczuk

**Affiliations:** 1 Department of Food Process Engineering, University of Life Sciences in Lublin, Głęboka Street 31, 20-612 Lublin, Poland; maciej.combrzynski@up.lublin.pl (M.C.); jakub.soja@up.lublin.pl (J.S.); tomasz.oniszczuk@up.lublin.pl (T.O.); 2 Student Research Circle of Food Engineering, Department of Food Process Engineering, University of Life Sciences in Lublin, Głęboka Street 31, 20-612 Lublin, Poland; michastaniak@gmail.com; 3 Department of Thermal Technology, University of Life Sciences in Lublin, Głęboka Street 31, 20-612 Lublin, Poland; beata.biernacka@up.lublin.pl; 4 Department of Inorganic Chemistry, Medical University of Lublin, Chodźki 4a, 20-093 Lublin, Poland; 5 Centre for Innovation and Research on Pro-Healthy and Safe Food, University of Agriculture in Krakow, Balicka 104, 30-149 Krakow, Poland; marek.gancarz@urk.edu.pl; 6 Faculty of Production and Power Engineering, University of Agriculture in Krakow, Balicka 116b, 30-149 Krakow, Poland; 7 Institute of Agrophysics Polish Academy of Sciences, Doświadczalna 4, 20-290 Lublin, Poland; 8 Institute of Agricultural Engineering, Wroclaw University of Environmental and Life Sciences, Chełmońskiego Street 37a, 51-630 Wrocław, Poland; magdalena.krecisz@upwr.edu.pl; 9 Toxicology Clinic, Faculty of Medicine, Medical University of Lublin, 100 Krasnik Avenue, 20-550 Lublin, Poland; jaroslaw.szponar@umlub.pl; 10 Clinical Department of Toxicology and Cardiology, Stefan Wyszynski Regional Specialist Hospital, 100 Krasnik Avenue, 20-550 Lublin, Poland

**Keywords:** functional food, cricket powder, extrusion-cooking, physical properties, antioxidant activity

## Abstract

Functional foods represent a new and thriving area of research. A significant direction of these studies is based on new products containing edible house cricket-derived additives. The aim of the presented studies was to determine the effect of using cricket powder (at 10% and 30% content) on the extrusion cooking parameters and the nutritional value, antioxidant activity, and selected physical properties of extruded potato-based snack pellets. The results suggest that house cricket powder is a promising functional ingredient. The processing efficiency and the physical and functional properties of the extrudates, including SME, WAI, WSI, bulk density, and mechanical durability, were affected by the addition of cricket powder, screw speed, and moisture content. Generally, higher levels of cricket powder reduced processing efficiency and altered structural properties due to changes in composition, particularly the balance between protein, fiber, and starch. The addition of cricket powder significantly improved antioxidant activity (>94% of DPPH scavenging for 30% content of additive) and increased the total polyphenol content in the assessed samples in comparison to potato bases (212.3 and 21.7 μg GAE/g dry weight, respectively). These innovative snack pellets containing cricket powder could be an appealing option due to their potential health benefits.

## 1. Introduction

The Food and Agriculture Organization (FAO) of the United Nations projects that by 2050, the global population will increase by approximately two billion, intensifying the demand for nutritious and sustainable food sources [1]. Among the strategies to meet these needs, the development of functional foods—products that provide physiological benefits beyond basic nutrition—has gained particular attention. One promising solution is the use of edible insects, which offer high-quality protein and bioactive compounds with potential health-promoting effects [2,3]. In the future, cricket powder-based food production could play a crucial role in combating climate change and ensuring the sustainable development of food systems.

Scientific research confirms a wide range of nutrients in edible insect species, with protein content up to 81.11 g/100 g [4]. One of the most widespread and industrially exploited species is the house cricket, *Acheta domesticus* [5]. These insects are valuable because they are rich in proteins (55.0–73.0 g/100 g dry matter) and lipids (4.3–33.4 g/100 g dry matter). They also contain macro- and micronutrients such as calcium, magnesium, potassium, sodium, phosphorus, zinc, iron, copper, and manganese, as well as vitamins B, A, C, D, E, and K [5]. Cricket protein has also been noted as a potential source of bioactive peptides. In addition, cricket protein is complete, meaning it contains all the essential amino acids that the human body needs from food. Crickets also contain chitin, which promotes digestive health, improves digestion, and regulates cholesterol levels. Several studies have highlighted that crickets contain polyphenols, which contribute to their antioxidant and anti-inflammatory activities. These compounds can scavenge free radicals and protect cells from oxidative damage, which is significant for the prevention of chronic and lifestyle-related diseases. Moreover, the antioxidant potential of insect-derived ingredients suggests that their inclusion in foods could improve both nutritional quality and product stability [6].

Potato snacks often have a low protein content and weak antioxidant properties, but the addition of cricket powder can address these issues. The polyphenol content of ingredients plays a key role in determining the antioxidant potential and quality of extruded products. Polyphenols can act as natural antioxidants and improve the oxidative stability. The study conducted by Quinteros et al. [7] found high polyphenol levels and antioxidant and anti-inflammatory activity in cricket product, with FRAP values of 70,034 µmol Trolox equivalents/g. Other researchers analyzed the protein concentrate derived from the cricket *Gryllus assimilis*, finding that the raw powder contained approximately 45.8% protein and the concentrate approximately 71.2%. [8]. Consequently, an increasing number of studies are being conducted on the utilization of cricket powder in food products. Cricket powder has been used as a low-carbohydrate and high-protein substitute for regular powder in muffin baking [9]. Other findings confirm that cricket powder can be used to produce protein-enriched bread [10]. It has been demonstrated that incorporating 10% cricket powder does not compromise the quality of the final product and results in a substantial enhancement of the nutritional value of wheat bread. Furthermore, fortification with insect powder has been shown to enhance the amino acid score (AAS) for lysine from over 40% to nearly 70% compared to wheat bread [11]. Legislative frameworks governing the use of cricket powder in food products have been established, with the European Union Commission Implementing Regulations for trading formally sanctioning the use of cricket powder derived from *Acheta domesticus* on 3 January 2023 [12]. Regulation 2023/5 is a consequence of the European Food Safety Authority (EFSA) opinion of May 2022 [13]. The addition of cricket powder to a wide range of food products, including pasta, sauces, baked goods, biscuits, chocolate, meat analogs, and other food products, including snacks, is permitted. It is imperative that the addition of cricket powder is explicitly indicated on the relevant food product packaging [14]. Among the range of snacks available on the market, those produced using extrusion technology merit particular attention [15]. The extrusion process entails subjecting the biological material to a brief, elevated-temperature treatment (HTST), characterized by continuous agitation, heating, pressure, and shear forces. However, the interactions with proteins and starch during extrusion may alter product color, texture, and expansion properties. Polyphenol–protein and polyphenol–starch interactions can influence water absorption, dough viscosity, and matrix expansion, thus affecting the final product’s structure and sensory appeal [16]. Given these relationships, this study aims to evaluate the effect of cricket powder addition and modifying processing parameters on the polyphenol content, antioxidant activity, and selected physical properties of extruded snack pellets based on potato powder. The parameters analyzed included the free radical scavenging activity, total phenolic content, and sum of free phenolic acids. This study forms part of broader research investigating the utilization of cricket powder in the production of functional foods, utilizing a range of starch-based ingredients.

## 2. Results

### 2.1. Free Radical Scavenging Activity—DPPH Method

**Figure 1:**
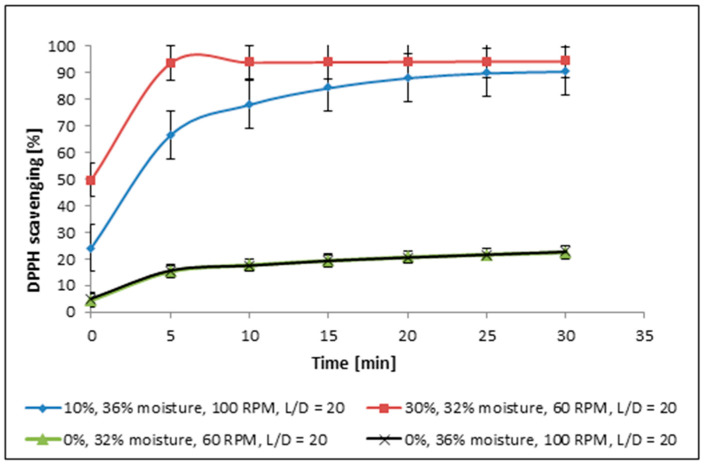
DPPH-scavenging activities obtained for functional food products enriched with 10% and 30% of cricket powder: moisture, RPM—screw rotation speed, and L/D—length-to-diameter ratio.

The results obtained for the DPPH method allowed us to determine the influence of extracts from functional food products enriched with 10% and 30% of cricket powder. In both cases, the extracts revealed a high ability to scavenge free radicals, which is highly desirable for food, especially functional food with high pro-health properties. The free radical scavenging increased with an increase in cricket powder. Results indicated that extracts from the food product with 10% of additive allowed it to scavenge approx. 90% of free radicals within 30 min, whereas a higher content of the additive (30%) allowed it to scavenge 94.4% at the same time (Figure 1). The differences are undoubtedly linked with the content of cricket powder; nevertheless, the differences are not significant.

More significant differences in antioxidant activity can be observed at the beginning of the experiment (Figure 1). Monitoring activity over a defined time period enables the identification of compounds or extracts that demonstrate superior performance, not only in their percentage of free radical scavenging but also in the time required to reach a high scavenging value. This approach also provides insights into the stability of the reaction over time. It is easy to notice the significantly higher free radical scavenging ability of sample with 30% of the additive in comparison to sample with 10% content of the powder. The differences explicitly indicate higher activity of the sample with a higher content of cricket additive. An additional sign of better activity is that it reaches a plateau faster.

**Figure 2:**
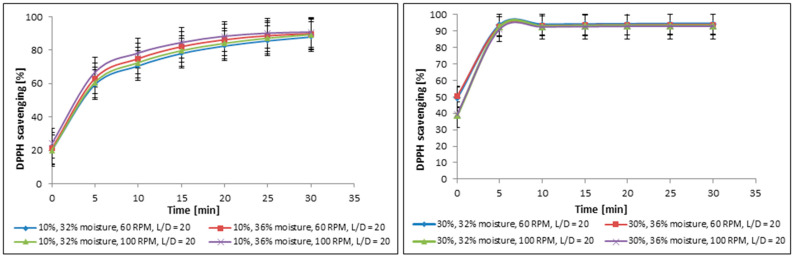
Impact of production parameters: moisture and screw rotation speed (RPM) on free radical scavenging ability of functional food products enriched with 10% and 30% of cricket powder; L/D—length-to-diameter ratio.

Besides the analysis of impact of additive on free radical scavenging activity, the production parameters used during sample preparation may also impact the biological property. The studies allowed us to analyze the influence of moisture and screw rotation speed (RPM) on their antioxidant activity (Figure 2).

In both cases, the tested production parameters did not exert a statistically significant impact on the antioxidant activity. For samples containing 10% cricket powder, the variation between the extreme values did not exceed 2.7%, while for those with 30% cricket flour, the difference was only 1.43%. Such small variations fall within the range of experimental error, confirming that the observed differences are not statistically meaningful. Although slightly higher antioxidant activity values were observed under specific production conditions (36% moisture and 100 RPM for the 10% additive, and 32% moisture and 100 RPM for the 30% additive), statistical analysis confirmed that these differences were not significant with respect to moisture content and screw rotation speed. Therefore, any apparent trends in antioxidant activity among the tested formulations should be interpreted cautiously, as the lack of statistical significance indicates that the observed variations may have arisen by chance and do not reflect a robust effect of the production parameters.

### 2.2. Total Content of Polyphenolic Compounds (TPCs) and Chromatographic Analysis of Free Phenolic Acids

The Folin–Ciocalteu method is an in vitro method that allows the determination of the approximate content of phenolic compounds in analyzed samples. It is commonly known that polyphenols are flagship antioxidants that largely determine the activity of the samples. The results of the study revealed that the amount of polyphenols increases as the level of cricket powder increases. The results of the study revealed that the amount of polyphenols in samples increases as the level of cricket powder increases (Table 1). In the case of 10% of cricket powder content, the TPC did not exceed 85 μg GAE/g dry weight (dw), whereas 30% addition of the powder caused a much higher TPC, equal to 212.3 μg GAE/g dry weight. In both cases the increase in polyphenols was significant in comparison to the potato base, for which the TPC did not exceed 23.1 μg GAE/g dry weight.

Similarly to antioxidant activity, the production parameters used for the reparation of the functional food product can have a crucial impact on their pro-health properties. Based on the test results presented in Table 1, both the percentage content of cricket powder and the production parameters affected the TPC value. The differences were equal to 17.3 μg GAE/g dw for 10% of cricket powder and 42.6 μg GAE/g dw for 30% of cricket powder content.

In the final step of the determination of polyphenolic compounds, the content of free phenolic acids was analyzed by chromatography for the sample without added cricket powder and for the sample enriched with 30% cricket powder with the highest TPC (30% added, 36% moisture, 60 RPM). The following phenolic acids were identified in the pellets without additive (Table 2): gallic, chlorogenic, caffeic, vanillic, and ferulic. In pellets containing 30% cricket powder, the following phenolic acids were identified: gallic, chlorogenic, caffeic, syringic, 4-OH-benzoic, *p*-coumaric, sinapic, and ferulic.

### 2.3. Principal Component Analysis (PCA)

**Figure 3:**
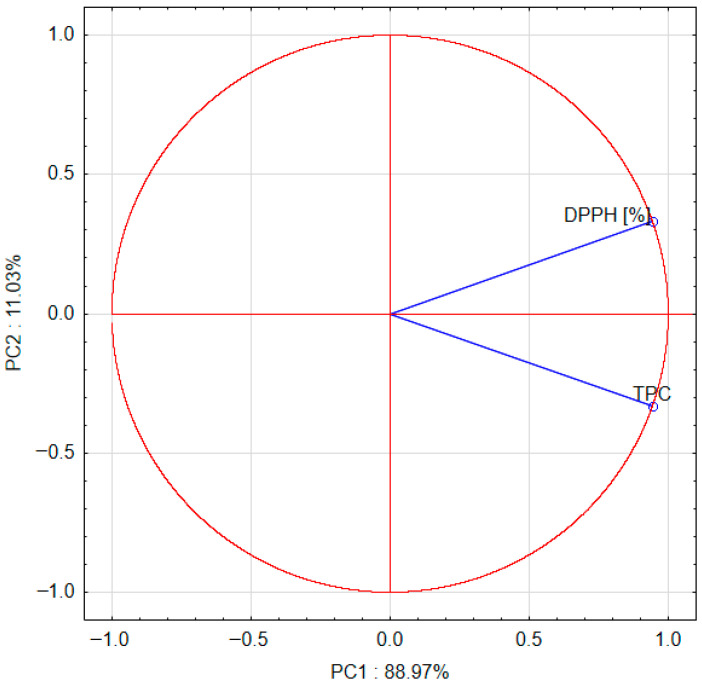
Projection of variables: DPPH (2,2-diphenyl-1-picrylhydrazyl) and TPC (total phenolic content) on the PC1 and PC2 score plot.

The PCA shows that the first two principal components, PC1 and PC2, describe the system variability in 100%. Both parameters tested have the greatest impact on the system variability (Figure 3). The parameters tested are weakly and positively correlated.

The PCA shows that the first principal component, PC1, describes the differences between the use of the additive and the addition of cricket powder in 88.97% (Figure 3). Positive values of the PC1 principal component describe the results for 10% and 30% addition of cricket powder. Negative values of the PC1 principal component describe the results for the absence of this type of powder. The second principal component, PC2, describes the differences between 10% addition of cricket powder and 30% addition of cricket powder and the absence of this type of powder in 11.03%. Positive values of the PC2 principal component describe the results for 10% addition of cricket powder. Negative values of the PC2 principal component describe the results for the absence of this type of powder and for 30% addition of cricket powder.

**Figure 4:**
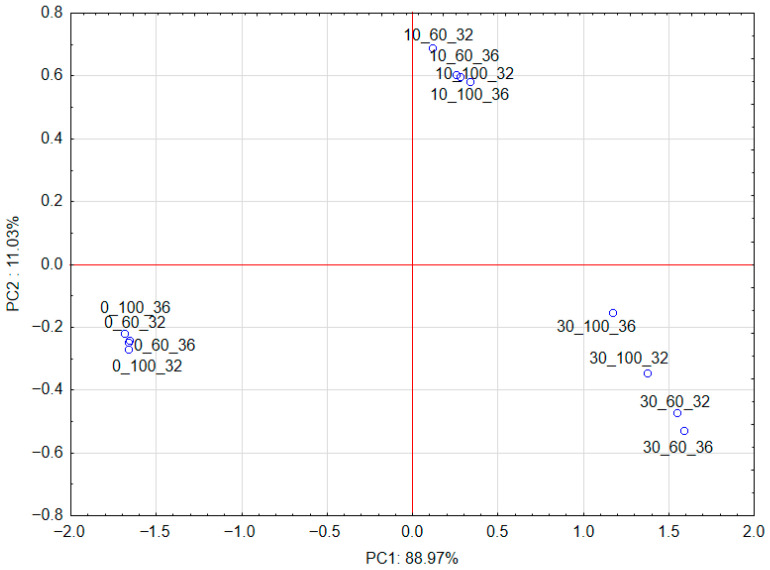
Projection of cases characterizing the level of cricket powder addition on the PC1 and PC2 loading plot.

The PCA (Figure 3 and Figure 4) also shows that DPPH is weakly but positively correlated with 10% addition of cricket powder, and TPC is positively correlated with 30% addition of cricket powder. These parameters are negatively correlated with powder without the addition of cricket powder.

### 2.4. Efficiency of the Extrusion Cooking Process

Increasing speed from 60 to 100 RPM significantly improved efficiency (by up to 75%) regardless of other parameters. The highest efficiency (36.16 kg/h) was recorded for the mixture without cricket powder at 36% moisture and 100 RPM (Table 3). The addition of house cricket powder reduced efficiency, particularly at the highest level (30%), likely due to increased mixture viscosity. At a lower speed (60 RPM), the impact of the powder addition was less pronounced and efficiency remained relatively stable at around 20–21 kg/h. An increase in moisture from 32% to 36% positively impacted efficiency, but only with a low or zero amount of house cricket powder. The results confirm the dominant influence of screw speed and highlight the need for balancing the powder addition and moisture content for optimal processing.

### 2.5. Energy Consumption of the Extrusion Cooking Process

The SME (specific mechanical energy) value was mainly affected by the addition of house cricket powder and screw speed, while moisture content had less impact (Table 3). The highest energy consumption, 0.053 kWh/kg, was observed with a 10% house cricket powder addition and lower screw speed, suggesting increased mechanical resistance due to the higher viscosity of the mixture. As the screw speed increased, there was a strong decrease in SME; for the mixture without cricket powder, values dropped from 0.038 to 0.016 kWh/kg. Energy consumption was lowest at 30% cricket powder, reaching just 0.010 kWh/kg. This may be due to reduced internal friction, likely resulting from lower starch content and a looser mixture structure. The effect of moisture content was variable—in some combinations, an increase in moisture led to a reduction in SME (e.g., 10% house cricket powder, 100 RPM), but at a low screw speed, this effect was less predictable.

### 2.6. Water Absorption Index (WAI)

House cricket powder and moisture content were the main factors affecting WAI, while screw speed had less influence. For the control mixture, WAI increased with both higher moisture and higher screw speed, reaching a maximum of 4.07 g/g. The addition of 10% house cricket powder caused the largest differences—at 32% moisture, WAI increased to 4.72 g/g, while at 36% moisture, it dropped sharply to 2.81 g/g, which could indicate a change in the starch structure and the presence of insect protein. For the 30% cricket powder addition, a strong decrease in WAI was observed, especially at 100 RPM and 36% moisture, where the lowest value in the entire set was recorded—2.75 g/g. This may suggest limited absorption capacity due to the dominance of proteins and fiber over starch components. The effect of screw speed was noticeable mainly at 0% and 10% addition, where higher speed promoted a higher WAI at low moisture. For the 30% cricket powder addition, this effect disappeared, and WAI remained at a lower level, ranging from 2.75 g/g to 3.52 g/g.

### 2.7. Water Solubility Index (WSI)

The WSI value was strongly influenced by the house cricket powder addition; the impacts of screw speed and moisture content were secondary. In the control sample, a noticeable increase in WSI was observed with higher moisture—from 8.24% to 17.21% (60 RPM) and from 7.54% to 18.35% (100 RPM), indicating intensive starch degradation. Adding 10% house cricket powder led to a significant decrease in WSI, with values consistently around 3.6% to 3.8%, regardless of the conditions. This suggests reduced solubility due to the presence of insect proteins and fiber. The lowest values were observed with a 30% house cricket powder addition, reaching as low as 2.57% at 100 RPM and 32% moisture, which may indicate strong inhibition of starch degradation and greater structural stability of the extrudate. In this group, WSI values increased moderately with moisture, reaching a maximum of 6.14% (60 RPM, 36% moisture), but they remained significantly lower than in the samples without additives.

### 2.8. Bulk Density of the Snack Pellets

The bulk density of the extrudates was strongly dependent on the house cricket powder addition, as well as the screw speed and moisture content. For the control sample, the values remained high (from 384.64 to 425.23 kg/m^3^) with only slight variations depending on the process conditions. The addition of 10% house cricket powder led to a decrease in density, especially at lower moisture and speed (328.23 kg/m^3^), which may indicate an increased expansion of the structure. The lowest values were observed for the 30% house cricket powder addition, reaching 262.03 kg/m^3^. This effect could be due to the presence of proteins and fiber, which limit the starch’s ability to form a compact structure. The screw speed positively influenced the density for both the 10% and 30% additions. Increasing the speed from 60 to 100 RPM raised the density, which may be associated with better thickening of the particles.

### 2.9. Durability of the Snack Pellets

The highest mechanical durability values of the pellets were recorded in the samples without house cricket powder addition, ranging from 98.14% to 98.71%, depending on the process conditions. The addition of 10% house cricket powder reduced durability to values ranging from 96.43% to 97.41%, while with 30% addition, the durability decreased further to 95.21% to 96.42%. The lowest result was observed for the variant with 30% addition, 60 RPM, and 32% moisture. Increasing the screw speed to 100 RPM did not significantly improve the durability in the mixtures with the additive. A slight but noticeable effect was observed with the increase in moisture from 32% to 36%, which generally improved durability, especially with the 30% addition. Mechanical durability decreased with an increase in cricket powder content, which could indicate a deterioration in the structural cohesion of the material. Nonetheless, durability values remained above 95% in all variants, indicating generally good mechanical resistance in the obtained extrudates.

The results for physical properties of snack pellets enriched with cricket powder are presented in Table 4.

## 3. Discussion

### 3.1. Free Radical Scavenging Activity, Total Phenolic Content and Chromatographic Analysis of Free Phenolic Acids

A growing body of research confirms the rationale, even the necessity, of including insects in a healthy and balanced diet [17]. Powder from house crickets contains many valuable nutrients and biologically active compounds and is gluten-free and can be consumed by people with a healthy lifestyle and athletes, as well as those with digestive problems and those suffering from celiac disease. A comparison of the properties of cricket powder with other cereals or pseudo-cereals (wheat, oats, or quinoa) showed that cricket powder with comparable technical and functional properties had a significantly higher protein (62.68–67.48%) and fat content (19.32–24.9%) and significantly higher antioxidant properties [6].

The obtained study results clearly indicate the high free radical scavenging activity of snack pellets enriched with cricket powder. A comparison of the activity and total phenolic content of the control samples (potato base) and the food containing the additive (10% and 30% cricket powder) revealed that the additive was responsible for both the free radical scavenging ability and the increased polyphenol content. It may be intriguing to consider the provenance of secondary plant metabolites, specifically polyphenols, in insect powder. Indeed, insects have evolved a sophisticated system for sequestering and metabolizing phenolic compounds from their diet. As early as the mid-20th century, scientific research had revealed the presence of phenolic compounds in the cuticles, wings, and digestive tracts of insects. The selective uptake of quercetin and kaempferol from host plants was observed by Burghardt et al. in the blue butterfly *Polyommatus icarus* [18]. It is now well established that during the process of sclerotization (the hardening of the insect exoskeleton), phenolic compounds are incorporated into the cuticular matrix in conjunction with structural proteins and chitin [19]. The presence of phenolic compounds in edible insects is subject to variation depending on the species, attributable to the consumption of different types of food and species [20]. As Nino et al. [20] observed, targeted phenolic compounds in *A. domesticus* were characterized in a similar manner. Over the course of the study, nine acids were determined, namely, gallic, chlorogenic, caffeic, syringic, 4-OH-benzoic, *p*-coumaric, sinapic, and ferulic, as well as 2-hydroxybenzoic. In a study by Ferreres et al. [21], phenolic acids (ferulic, sinapic, and *p*-coumaric acids) were identified in extracts from larvae of the cabbage white butterfly (*Pieris brassicae*) reared on turnip. Moreover, ferulic and sinapic acids have been detected in larvae of the white butterfly reared on Portuguese cabbage [22]. An investigation into the total phenolic content of unprocessed edible beetles (*Eulepida mashona*) revealed a content of 0.08 mg GAE/100 g sample [23]. In a separate study, the total phenolic content of ground edible crickets (*Henicus whellani*) was found to be 0.77 g GAE/100 g, which was lower than the values presented in the current study for *A. domesticus* [24]. Hirayama et al. [25] determined the contents of kaempferol and quercetin glycosides from the cocoon of the white caterpillar (*Rondotia menciana*), which was exclusively fed mulberry leaves. However, these compounds were not identified in the host plant, suggesting that insects are capable of metabolizing flavonoids for further incorporation into the cocoon. Ruggeri et al. [26] investigated the antioxidant activity in spray-dried cricket powder. The researchers recorded an increase in free radical scavenging activity, as well as activity in chelating iron ions and EDTA equivalents. The results indicated the release of bioactive compounds from cricket samples, leading to increased antioxidant activity.

An experiment conducted by the authors demonstrated that innovative potato snack pellets enriched with as little as 10% addition of cricket powder exhibited significantly higher polyphenol content and free radical scavenging activity in comparison to a sample without this addition. It is well established that polyphenols are a prominent class of antioxidants, exhibiting a diverse range of biological activities, including enzyme inhibition, metal chelation, anticancer, antifungal, anti-viral, and other properties [27].

While the observed increase in antioxidant activity in snacks with the addition of cricket powder, as indicated by the DPPH radical scavenging capacity and total phenolic content (TPC), provides strong evidence of its bioactive potential, the underlying mechanisms responsible for this effect remain underexplored. Several classes of compounds inherent to insects—namely, polyphenols, chitin, and protein-derived peptides—may each contribute distinctly to the antioxidant profile of cricket powder [5]. Polyphenols are well-known for their ability to donate hydrogen atoms or electrons, neutralizing free radicals and thereby exhibiting strong antioxidant properties. Increases in total phenolic content after processing cricket powder suggest that these low-molecular-weight compounds could be partially responsible for the improved DPPH activity.

However, while TPC assays are often used as a proxy for polyphenol concentration, they can also detect non-phenolic reducing agents and therefore may overestimate the true polyphenol content. Chitin, a structural polysaccharide abundant in the exoskeletons of insects, has been reported to exhibit moderate antioxidant activity, particularly when deacetylated to form chitosan. The antioxidant mechanisms of chitin and its derivatives include metal ion chelation, the scavenging of hydroxyl and superoxide radicals, and the inhibition of lipid peroxidation. However, native chitin in cricket powder is relatively insoluble and may not contribute significantly to radical scavenging in DPPH assays, which typically involve soluble antioxidants. Nonetheless, during processing (e.g., milling, enzymatic hydrolysis, fermentation), partial deacetylation or degradation of chitin may occur, potentially enhancing its bioavailability and antioxidant function. The extent to which chitin contributes depends on both its physicochemical form and the processing conditions applied to the cricket powder [6].

Proteins in cricket powder are another likely source of antioxidant activity. During digestion, fermentation, or processing (e.g., enzymatic hydrolysis), bioactive peptides can be released. These peptides may contain amino acid residues such as tyrosine, tryptophan, cysteine, and histidine, which are known to donate electrons or hydrogen atoms and chelate pro-oxidant metal ions. Studies on insect protein hydrolysates have demonstrated significant antioxidant properties, often rivaling synthetic antioxidants. Moreover, the Maillard reaction products formed during heat processing (e.g., roasting or baking with cricket powder) may also contribute to antioxidant capacity. These products can interact with both proteins and phenolic compounds, complicating the attribution of antioxidant effects to a single component class [6].

The pro-health properties of crickets as additives to food were presented by Zielińska et al. [28], who decided to determine the biological value of muffins enriched with edible insect powder. Their studies revealed that the total phenolic content and antioxidant capacity against free radicals (e.g., DPPH) increased correspondingly as the percentage of insect powder in the muffins increased, as observed in our study. The authors also noted that this fortification reduced the predicted glycemic index, making the inclusion of insect powder beneficial for individuals with conditions such as diabetes, insulin resistance, and obesity [29]. Kowalczewski et al. [30] conducted a study on gluten-free bread enriched with cricket powder, in which they also found that the addition of cricket powder enhanced the measured antioxidant activity. Suh et al. [29] investigated the antioxidant properties of extracts obtained from *A. dichotoma*, reporting moderate scavenging activity that varied with the extraction solvent, with the methanolic extract showing the lowest IC50 (0.119 mg/mL). Conversely, aqueous extracts from *H. parallela* demonstrated significant antioxidant activity (IC_50_ of 1.45 mg/mL) [31]. Additional studies have highlighted a correlation between total phenolic content and DPPH scavenging activity for *T. molitor* and *A. domesticus* extracts, showing a clear relationship between higher polyphenol content and increased DPPH scavenging capacity [32], as in our study. High activity in FRAP and ORAC assays was also obtained for aqueous extract cricket powder [33]. Authors evaluated the impact of thermal processing on the antioxidant activity of the product. Assays revealed that thermally threated cricket powder showed significantly diminished electron transfer, leading to antioxidant activity improvement. Considering the positive impact of temperature treatment as well as high antioxidant activity and TPC of the analyzed functional food, it can be assumed that cricket powder is a valuable additive, significantly improving food nutritional and pro-health values.

A significant element of the study was the analysis of the effect of production parameters on the antioxidant potential of the samples studied and on the content of active substances. The findings of the present study suggest that extrusion conditions exert a substantial influence on the content of polyphenolic compounds in the pellets. This observation was replicated by other researchers during their own experimental investigations. During the thermal processing of cricket powder-based products, significant changes in physicochemical properties and biological activity occur [33]. The extrusion texturization process was investigated for its potential in the production of meat analogs comprising soy protein isolate and cricket powder. The results demonstrated the potential to produce fibrous meat analogs with high anisotropic indices using 30% low-fat cricket powder [34]. Furthermore, the incorporation of edible insects (5 and 10%) into extruded corn-based snacks has been shown to provide a viable alternative to market snacks, as the product retains adequate physicochemical properties while exhibiting a significantly higher protein content [35]. Other researchers have confirmed that it is possible to obtain corn snacks enriched with 12.5 and 15.0% cricket powder as a source of protein, but in order to maintain proper sensory properties (color, aroma, taste, and mouthfeel), the use of 7.5% cricket powder is recommended [14]. The replacement of a proportion of wheat flour with cricket powder in the preparation of chapatti has been proposed in other studies, which noted that the amount of cricket powder added should not exceed 5%, as a higher proportion has been shown to deteriorate the texture of the product [36]. Another study evaluated the effect of replacing soy flour with cricket powder and observed that this supplementation improved the nutritional value and in vitro digestibility of the protein but reduced the viscosity, bulk density, water absorption capacity, and solubility of the powder in water, making it suitable for feeding children [37].

The consumption of products made from cricket powder from *Acheta domesticus* crickets can have many health benefits, including high free radical scavenging activity and significant levels of polyphenolic compounds. Combined with the environmental benefits of reduced consumption of natural resources, insect-based food production will become a necessity in the future to meet the challenges of a growing population and climate change and to contribute to the health of society.

### 3.2. Extrusion Cooking Parameters

The extrusion cooking process efficiency was primarily dependent on the screw speed and to a lesser extent on the moisture content and the addition of house cricket powder. This is similar to the study by Lisiecka and Wójtowicz [38], where the highest efficiency (above 34 kg/h) was achieved with a low vegetable addition (2.5–5%) and higher screw speed (120 RPM). The highest efficiency value (36.16 kg/h) in the present experiment occurred without the addition of house cricket powder and at 100 RPM (Table 3). In both cases, an increase in the addition (vegetables or insect powder) led to a decrease in productivity, which can be explained by the increase in moisture and viscosity, making it more difficult for the material to flow through the extruder. In contrast with the observations of Matysiak et al. [39], where moisture content had a greater impact on extrusion cooking process efficiency than screw speed, an increase in screw speed from 60 to 100 RPM emerged as the primary factor enhancing productivity in the present study. According to the findings of both studies, an increase in moisture from 32% to 36% positively influenced productivity, especially with a lower level of the additive. This effect may be attributed to the reduction in viscosity of the mixture, which facilitated its flow through the extruder barrel.

The study by Mitrus et al. [40] found that SME values ranged from 0.014 to 0.124 kWh/kg, being influenced by the moisture content of the mixture and the speed of the screw. In most cases, higher moisture levels reduced energy consumption. A similar trend was observed in the present study, where increasing the moisture content from 32% to 36% resulted in lower SME values, particularly at higher levels of house cricket powder. However, in contrast with the results reported by Mitrus et al. [38], where an increase in screw speed sometimes resulted in higher SME values, raising the speed from 60 to 100 RPM in this study consistently reduced energy consumption, regardless of the mixture composition. This difference may be due to the specific structure of the insect-based material, which likely resulted in lower mechanical resistance during the extrusion cooking process.

In contrast with the study by Lisiecka and Wójtowicz [41], where SME values ranged from 0.08 to 0.25 kWh/kg and consistently increased with rising screw speed, regardless of the type and amount of plant-based additives, the present research revealed an opposite trend. Increasing the screw speed from 60 to 100 RPM resulted in a decrease in SME, for example, from 0.048 to 0.026 kWh/kg in control samples without insect powder. Moreover, a higher proportion of house cricket powder was associated with reduced mechanical energy consumption, with the lowest SME value (0.020 kWh/kg) recorded at 30% addition. This may be related to the lower starch content and higher protein proportion, which could facilitate material flow within the extruder. Despite the differing trends, both studies confirmed that screw speed has a significant influence on SME, although the direction of this effect appears to depend on the nature of the added ingredients—plant-based versus insect-based.

### 3.3. Selected Physical Properties of Extruded Pellets

According to the findings of Wójtowicz et al. [42], increasing the proportion of house cricket powder led to a reduction in bulk density. A similar trend was observed in the present study—at a 30% addition of house cricket powder, the bulk density dropped to 262.03 kg/m^3^, with the lowest values also recorded at higher moisture levels (Table 4). Both studies confirmed that a higher share of cricket powder results in a looser extrudate structure, likely due to the weakening of the starch network caused by the presence of proteins and fiber. In the study by Dushkova et al. [43], the highest bulk density was obtained with a high moisture level and the addition of goji berries, and the effect of moisture was considered more significant than that of the additive itself. Herein it was also observed that the moisture content of the raw material influenced bulk density. However, the most substantial changes in bulk density were caused by the level of house cricket powder addition—especially at 30%, where bulk density decreased to 262.03 kg/m^3^. Unlike the observations of Dushkova et al. [43], in this study, increasing moisture was generally associated with a decrease in bulk density, which may be a result of intensified expansion under conditions of lower starch content.

Consistent with the findings reported by Soja et al. [44], the highest mechanical durability values were recorded in the control samples—without any additive—regardless of screw speed. In both studies, the addition of a component (pomace or house cricket powder) led to a gradual decline in durability as the inclusion level increased. In the present study, durability dropped to a minimum of 95.21% at a 30% addition and 60 RPM, which was a more noticeable reduction than in Soja’s work, where the lowest values at 30% addition remained above 99%. This difference may be attributed to variations in the fiber and protein composition of the respective additives. In the study by Mitrus et al. [40], the mechanical durability of extrudates remained at a very high level (99.06–99.88%), and the influence of processing parameters and the addition of fresh broccoli was minimal. In contrast to these findings, the present study observed a more pronounced reduction in durability with increasing levels of house cricket powder—down to 95.21% at a 30% inclusion rate—suggesting that the raw material composition had a greater impact on the mechanical structure of the product. Nevertheless, the durability of all tested variants remained high, indicating good resistance of the pellets to damage.

In the study by Wani and Kumar [45], the highest WAI value was obtained in control samples without the addition of fenugreek seed powder and fenugreek leaf powder, while the inclusion of 1% significantly reduced the index. Similarly, in the present study, the addition of house cricket powder also led to a decrease in WAI (Table 4). This effect was particularly pronounced at a 30% inclusion level and 36% moisture content, where the lowest WAI value—2.75 g/g—was recorded. In the study by Yagci et al. [46], WAI values consistently decreased with increasing levels of powdered tomato pomace. The authors attributed this to the reduced availability of structures capable of water interaction and a lower number of hydrophilic groups. A similar effect was observed in the present study, where increasing the proportion of cricket powder to 30% resulted in a WAI drop to 2.75 g/g. This reduction may be explained by the dominance of proteins and fiber over starch, which limits water absorption. Both studies confirm that ingredients high in non-starch components negatively affect the water absorption capacity of extruded products.

In the study by Pardhi et al. [47], increasing moisture content led to a reduction in the WSI of extrudates, which the authors attributed to reduced starch shearing under higher hydration conditions. A similar trend was observed in the present study for samples containing cricket powder, where higher moisture levels resulted in lower WSI values compared to the control (Table 4). The lowest WSI value (2.57%) was recorded at 30% cricket powder, 32% moisture, and 100 RPM. In contrast to Pardhi’s findings, the effect of screw speed in this study was less pronounced. Instead, the main factor limiting solubility appeared to be the formulation, particularly the presence of insect-derived proteins and fiber. Combrzyński et al. [48] observed that low WSI values were associated with the presence of fresh alfalfa sprouts—particularly at 30% addition, 60 RPM, and 32% moisture—which the authors attributed to high fiber content and low starch levels, hindering ingredient integration. A similar effect was noted in the present study, where the lowest WSI (2.57%) was also recorded at 30% house cricket powder, 32% moisture, and 100 RPM. This may suggest comparable mechanisms of reduced solubility linked to the presence of non-starch components.

## 4. Materials and Methods

### 4.1. Materials Used in the Production of Pellets

The extrusion cooking production process for snack pellets enriched with house cricket powder was meticulously developed and experimentally validated in the laboratories of the Department of Process Engineering at the University of Life Sciences in Lublin in 2021.

The preparation of the snack pellets was based on specifically designed formulations. The insect powder used in the study, containing powdered house cricket (*Acheta domesticus*) (a commercially available product), was sourced from SENS Foods (London, UK). According to the manufacturer’s data, cricket powder contains the following: moisture—2.5%, protein—70.0%, fat—17.0% (of which saturated fat—5.2%), fiber—9.3%, carbohydrates—0.5%; energy value—1939 kJ/463 kcal (per 100 g dw).

Additional ingredients, including premium-quality potato starch, potato flakes, rapeseed oil, beet sugar, and table salt, were procured from local suppliers. The inclusion of insect powder aimed to fortify the snack pellets with bioactive components such as B-group vitamins (B12, B2, B5), which are often limited in plant-based diets, as well as protein, beneficial fatty acids (including omega-3 and omega-6), and essential micronutrients such as iron, zinc, calcium, magnesium, phosphorus, and potassium. A control sample without the addition of insect powder was also prepared, allowing for a comparative analysis of the impact of house cricket powder incorporation on the structural, physical, and functional characteristics of the final product.

### 4.2. Preparation of Mixtures and Extrusion Cooking Process

**Figure 5:**
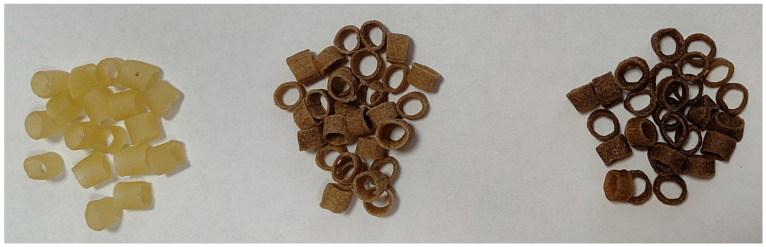
Pellets with 0, 10 and 30% (from left) of cricket powder.

Experimental formulations were developed, incorporating edible insect powder at three inclusion levels: 0%, 10%, and 30% (Figure 5) of the total ingredient weight (Table 5). These concentrations were selected based on preliminary tests and findings from the literature, aiming to achieve nutritional enhancement without compromising the sensory attributes or texture of the final product. Higher levels (>30%) were avoided due to potential processing issues such as extrusion instability and inconsistent product quality.

All raw materials were homogenized using a 0.5 mm mesh sieve to ensure uniform distribution and consistent moisture. The prepared blends were then stored under refrigeration for 24 h to allow moisture equilibration. The moisture content was adjusted to two target levels—32% and 36%—based on prior research and technological feasibility, with the aim of optimizing extrusion performance and product characteristics. Following adjustment, samples were again refrigerated for 24 h to ensure homogeneity.

Moisture levels were verified using a high-precision MA50R moisture analyzer (Radwag, Radom, Poland; accuracy ±0.001%). When needed, water was added to meet the desired moisture targets, facilitating assessment of how moisture content influences extrusion behavior and end-product quality.

Extrusion was carried out using a single-screw extruder EXP-45-32 (Zamak Mercator, Skawina, Poland) with an L/D ratio (length-to-diameter ratio) of 20. To evaluate the effect of screw speed, the process was conducted at two rotation rates: 60 RPM and 100 RPM. Processing temperatures in the extruder barrel ranged from 44 °C to 68 °C, conditions selected to preserve thermolabile bioactive compounds (Table 6).

The extrudate was shaped through a 25 mm × 0.6 mm ring die, producing strands that were cut into rings (approx. 2 mm diameter, 1.5 mm thickness). These were dried in a laboratory convection dryer at 40 °C for 12 h to reach a final moisture content of approximately 9%, ensuring microbial stability and desirable texture. Dried samples were sealed in airtight foil pouches until further analysis.

### 4.3. Efficiency of the Extrusion Cooking Process

To assess the performance of the extrusion process, a detailed quantitative evaluation was conducted on the material emerging from the forming die. This analysis involved systematically weighing the extrudate at consistent 30 s intervals under strictly controlled conditions. Throughout the duration of the experiment, all operational parameters—including feed rate, temperature, and equipment settings—were kept constant to ensure the reliability of the collected data and to isolate the variable of interest. Each data point was recorded in triplicate to minimize random variability and enhance both the precision and reproducibility of the results. A high-accuracy electronic stopwatch (model DS-788, Yakudo, Tokyo, Japan) was employed for timekeeping, while a digital timer was used to log the time intervals with precision. The mean mass of the extrudate collected during the test was subsequently calculated and utilized as a key metric for evaluating process efficiency. This average was regarded as representative for each specific combination of process settings, thereby allowing for meaningful comparisons across different test conditions. The adopted methodology was based on the procedure developed by Matysiak et al. [39], who introduced a comparable approach for assessing extrusion efficiency in blends containing potato powder. In the current study, this framework was adapted for extrudates incorporating house cricket powder, facilitating a reliable comparative analysis of how various processing parameters influence production efficiency.

Efficiency calculations were performed using the following expression:

(1) $$Q=\frac{m}{t}\left(kg/h\right)$$

where Q represents the extrusion process efficiency in kilograms per hour, m denotes the mass of the extruded product (kg), and t is the total duration of the measurement (h).

### 4.4. Energy Consumption of the Extrusion Cooking Process

Throughout the extrusion cooking process, active power consumption was systematically tracked via a built-in wattmeter, incorporated into the extruder’s control system. The device continuously collected real-time data on motor performance—such as voltage, current intensity, and load—providing a reliable foundation for subsequent energy efficiency assessments. Utilizing the motor’s technical specifications and the recorded process parameters, the mechanical energy demand per unit mass of processed material was determined. This value, referred to as SME, is a key indicator of the energy efficiency of the extrusion cooking process. The calculations were carried out in accordance with the method proposed by Matysiak et al. [39], which accounts for both the motor’s power input and the throughput of the extrusion process.

To convert the recorded data into SME values, a mathematical formula was applied to estimate the amount of mechanical energy transferred to the raw material during processing:

(2) $$SME=\frac{n}{n_{m}}\times \frac{O}{100}\times \frac{P}{Q}(kWh/kg)$$

where

SME—specific mechanical energy (kWh/kg);
n—screw rotational speed (RPM);
n_m_—maximum screw speed of the extruder;
O—motor load (%);
P—rated motor power as indicated on the control panel (kW);
Q—extrusion throughput (kg/h).

### 4.5. Bulk Density of Snack Pellets

The bulk density of the produced snack pellets was assessed following the method described by Combrzyński et al. [48], by calculating the ratio of sample mass to its corresponding volume. The mass of each sample was measured using a high-precision digital weight (WPS 210/C, Radwag, Radom, Poland), with an accuracy of ±0.001 kg. Volume determination was carried out using a cylindrical container with a capacity of 1 L. Each sample was measured in triplicate to ensure measurement reliability, with an estimated accuracy of ±0.01 kg/m^3^. The final bulk density for each sample was expressed as the arithmetic mean of the three measurements, which minimized random variability and enhanced result consistency.

Bulk density (BD) was calculated using the following equation:

(3) $$BD=\frac{m}{V}(kg/m^{3})$$

where BD represents bulk density (kg/m^3^), m is the sample mass (kg), and V is the volume of the container (m^3^).

### 4.6. Durability of Extrudates

The durability of the snack pellets was evaluated by measuring their resistance to mechanical stress induced by rotational forces within a sealed testing chamber. This assessment was conducted using a Pfost-type device, which applies a controlled level of kinetic energy to simulate mechanical impact on the samples. This technique enables precise evaluation of the material’s fracture resistance and facilitates the identification of structural weaknesses within the pellets. The resulting durability values serve as a useful metric for comparing the quality of different snack formulations or optimizing processing parameters, thereby supporting the production of high-quality, robust products with improved stability during handling, transport, and storage. The test procedure followed the methodology outlined by Wójtowicz et al. [42], ensuring both repeatability and compliance with recognized standards. Each sample was tested in triplicate, and the average of the three trials was calculated to provide a more reliable representation of mechanical strength.

Durability (D) was determined using the following formula:

(4) $$D=\frac{m_{pt}}{m_{i}}\times 100\%\;(\%)$$

where D is the durability percentage, m_pt_ is the mass of the sample after testing (g), and m_i_ is the initial sample mass (g).

### 4.7. Water Absorption Index of Snack Pellets

The Water Absorption Index was determined according to the method described by Lisiecka and Wójtowicz [38], which assesses the water-binding capacity of the examined extrudates. For the analysis, 0.7 g of ground sample was thoroughly mixed with 7 mL of distilled water. The mixture was continuously stirred for 20 min to ensure uniform hydration of the particles. Following the hydration phase, the suspension was centrifuged at 15,000 RPM for 10 min using a Digicen 21 centrifuge (Labsystem, Kraków, Poland). After centrifugation, the supernatant was carefully decanted, and the remaining gel was weighed using a high-precision analytical balance (WPS 210/C, Radwag, Radom, Poland) with an accuracy of 0.001 g.

Each sample was tested in triplicate to ensure measurement accuracy, and the final WAI value was calculated as the arithmetic mean of the three measurements. The index reflects the sample’s capacity to retain water following contact with a liquid and was calculated using the following equation:

(5) $$\;WAI=\frac{m_{g}}{m_{s}}(g/g)$$

where WAI represents the Water Absorption Index (g/g), m_g_ is the mass of the gel after centrifugation (g), and m_s_ is the mass of the dry sample (g).

### 4.8. The Water Solubility Index of Snack Pellets

The Water Solubility Index was determined following the procedure described by Lisiecka and Wójtowicz [38], as an extension of the Water Absorption Index (WAI) analysis. After centrifugation and removal of the supernatant, the liquid phase was subjected to evaporation under controlled laboratory conditions. Drying was carried out in a chamber dryer (SLW 53 STD, Pol-Eko Aparatura S.J., Wodzisław Śląski, Poland) at a constant temperature of 110 °C until complete water removal was achieved.

The remaining solid residue—representing the water-soluble fraction—was weighed using a high-precision analytical microbalance (WPS 210/C, Radwag, Radom, Poland) with a resolution of 0.001 g. Each sample was analyzed in triplicate to ensure accuracy and reproducibility. The final WSI value was calculated as the mean of the three measurements and expressed as the percentage of water-soluble substances relative to the initial sample mass, according to the following equation:

(6) $$WSI=\frac{m_{v}-m_{dv}}{m_{s}}\times 100\;(\%)$$

where WSI is the Water Solubility Index (%), m_v_ denotes the mass of the container before drying (g), m_dv_ is the mass of the container after drying, including the solid residues (g), and m_s_ is the mass of the dry sample (g).

### 4.9. Preparation of Extracts—Ultrasound-Assisted Extraction

The extracts were prepared with use of an ultrasonic bath (Bandelin Electronic GmbH & Co. KG, Berlin, Germany) with the following parameters: temperature: 60 °C, an ultrasound frequency of 33 kHz, and a power of 320 W [16]. In order to obtain extracts, 4 g of milled extrudes was mixed with 80 mL of methanol (99.8%) and set in the ultrasonic bath for 40 min. The extraction time and temperature had been optimized in previous tests to avoid the degradation of thermolabile compounds. After this time, the extracts were filtered and a new portion (80 mL) of methanol (99.8%) was added to the remainder to repeat the extraction process. Both obtained portions of extracts were combined, evaporated to dryness, and dissolved in 10 mL of methanol (99.8%). The samples were examined towards their free radical scavenging activity, total phenolic content, and sum of free phenolic acids.

### 4.10. Free Radical Scavenging Activity—DPPH Method

In order to determine the free radical scavenging activity of the obtained extracts, the DPPH (2,2-diphenyl-1-picrylhydrazyl) method was used [49]. The studies were performed with the use of a UV-VIS spectrophotometer Genesys 20 UV-VIST (Thermo Scientific, Waltham, MA, USA). The following parameters were used: 517 nm wavelength, measurements every 5 min for 30 min, and calibration based on pure methanol. All measurements were repeated three times. The free radical scavenging activity was calculated with the use of the following formula:

(7) $$\%RSA=\left[\frac{A_{0}-A_{1}}{A_{0}}\right]\times 100$$

where %RSA is radical scavenging activity (%), A_0_ is the absorbance of the control, A is the absorbance of the sample.

### 4.11. Total Content of Polyphenolic Compounds (TPC) with Use of Folin–Ciocalteu Method

The measurement was performed with the use of the modified Folin–Ciocalteu (FC) method [49]. Namely, 200 μL of extract was mixed with 1.8 mL of distilled water. Afterwards, 200 μL of FC reagent was added, mixed, and left for 5 min. In the next step, 2 mL of 7% Na_2_CO_3_ was added, and the mixture was incubated for 60 min at 40 °C. Absorbance was measured with the use of the UV-VIS spectrophotometer Genesys 20 UV-VIS (Thermo Scientific, Waltham, MA, USA) at 760 nm. The amount of phenolics was expressed as μg gallic acid equivalents (GAE) per g of dry weight.

### 4.12. LC-ESI-MS/MS Analysis of Phenolic Acids

The determination of phenolic acid content was conducted in accordance with a method previously outlined by Olech et al. [13]. Standards of phenolic acids and LC-grade acetonitrile were purchased from Sigma–Aldrich Fine Chemicals (St. Louis, MO, USA). Gentisic, sinapic, and veratric acids were from ChromaDex (Irvine, CA, USA). LC-grade water was prepared using a Millipore Direct-Q3 purification system (Bedford, MA, USA). Experiments were conducted utilizing an Agilent 1200 Series HPLC system (Agilent Technologies, Santa Clara, CA, USA), which was connected to a 3200 QTRAP Mass Spectrometer (AB Sciex, Marlborough, CA, USA). This instrument was equipped with an electrospray ionization source (ESI). Both were controlled with Analyst 1.5 software (AB Sciex, USA), which was also used for data interpretation. The separations were carried out on a Zorbax SB-C18 column (2.1 × 100 mm, 1.8 mm particle size; Agilent Technologies, Santa Clara, CA, USA) at 20 °C. The gradient method was employed with mobile phases comprising water with 0.1% HCOOH (A) and acetonitrile with 0.1% HCOOH (B). The injection volume was 3 µL, the flow rate was 250 µL/min, and the gradient was as follows: as demonstrated in Figure 1, the initial rate of B is 25% at 0–2 min, increasing to 35% at 3–6 min. This then rises to 55% at 8–10 min and 75% at 12–16 min, finally returning to 25% at 19–25 min. The experimental setup comprised the operation of ESI in negative-ion mode within the following conditions: capillary temperature of 400 °C, curtain gas at 30 psi, nebulizer gas at 50 psi, and a source voltage of −4500 V for negative ionization mode. Triplicate injections were performed for each standard solution and sample. The identification of the analytes was accomplished by undertaking a comparison of the retention time and *m*/*z* values obtained by means of MS and MS2 with the mass spectra from the standards that had been subjected to testing under analogous conditions. The calibration curves obtained in MRM mode were used to quantify all analytes. The quantification of the identified phenolic acids was conducted on the basis of their peak areas, in comparison with a calibration curve that had been obtained using the corresponding standards.

### 4.13. Statistical Analysis

In order to determine the accuracy of the measurements, means and deviations were calculated, and other statistical analyses were performed in Statistica 12.0 (StatSoft, Kraków, Poland). ANOVA and the Tukey test were used to see if there were any differences (*p* < 0.05) between the means. The significance of differences (*p* < 0.05) between the means was noted with different letters. Principal component analysis was used to determine the relationships between the studied cases and parameters. Principal components analysis (PCA) was performed at the significance level of α = 0.05. The matrix of data used for the PCA statistical analysis of research results had three columns and 12 rows. The Cattel criterion was used to determine the number of principal components in the analysis in both cases, and the input matrix was automatically scaled. All measurements were performed in triplicate.

## 5. Conclusions and Perspectives

The study confirmed that enriching a functional food product with house cricket powder significantly enhances its antioxidant properties, primarily due to increased polyphenol content and free radical scavenging activity. Both the DPPH and TPC analyses showed a clear correlation between higher cricket powder content and stronger antioxidant effects, with 30% addition resulting in the most pronounced activity. Importantly, while production parameters (moisture and screw speed) had minimal impact on DPPH activity, they influenced polyphenol content more noticeably, indicating their role in optimizing bioactivity. Chromatographic analysis revealed a broader spectrum of phenolic acids in samples with 30% cricket powder, further supporting the contribution of cricket-derived compounds to antioxidant capacity. The processing efficiency, as well as physical and functional properties of the extrudates—including SME, WAI, WSI, bulk density, and mechanical durability—were all affected by cricket powder addition, screw speed, and moisture content. Higher levels of cricket powder generally reduced the processing efficiency and altered structural properties due to changes in composition, particularly the balance between protein, fiber, and starch. Despite a slight reduction in mechanical durability and structural density with increased cricket content, all extrudates maintained acceptable technological quality. The findings demonstrate that house cricket powder is a promising functional ingredient that not only enhances the health-promoting properties of food but also performs well under various processing conditions.

To complement the nutritional and functional advantages of cricket powder, its environmental sustainability stands out when compared to conventional animal protein sources. A brief life-cycle analysis highlights several key advantages. Crickets emit significantly fewer greenhouse gases than ruminant livestock. Moreover, crickets can be farmed vertically and require minimal land area. Additionally, they can be fed on organic side streams, which supports circular economy principles and reduces overall waste. These environmental benefits—coupled with its functional and nutritional properties—make cricket powder a compelling ingredient for sustainable food systems.

To ensure product shelf life and consumer acceptance, the optimal formulation must strike a balance between nutritional value, processing performance, and textural properties. However, this study did not address the microbiological safety or stability of the snacks, nor their consumer acceptance. These aspects are areas for future research.

## Figures and Tables

**Table 1:** Total phenolic content obtained for functional food samples enriched with 10% and 30% of cricket powder (n = 3; ±SD).

Cricket Powder Content	Production Parameters	Total Phenolic Content [μg GAE/g dw]
10%	32% moisture, 60 RPM	67.5 ± 2.7 ^b^
10%	36% moisture, 60 RPM	81.0 ± 0.6 ^c^
10%	32% moisture, 100 RPM	79.5 ± 9.4 ^bc^
10%	36% moisture, 100 RPM	84.8 ± 4.0 ^c^
30%	32% moisture, 60 RPM	207.3 ± 65.3 ^d^
30%	36% moisture, 60 RPM	212.3 ± 12.9 ^d^
30%	32% moisture, 100 RPM	191.0 ± 14.7 ^d^
30%	36% moisture, 100 RPM	169.7 ± 19.3 ^d^
0%	32% moisture, 60 RPM	19.1 ± 0.2 ^a^
0%	36% moisture, 60 RPM	21.7 ± 0.5 ^a^
0%	32% moisture, 100 RPM	23.1 ± 0.3 ^a^
0%	36% moisture, 100 RPM	21.8 ± 0.5 ^a^

^a–d^—means indicated with the same letters in columns do not differ significantly at α = 0.05.

**Table 2:** LC-ESI-MS/MS analytical results for phenolic acids investigated in samples (n = 3; ±SD). Compounds confirmed by comparison with authentic standards.

Compound	T_R_ (min)	Pellets Without Cricket Powder [mg/100 g dw]	Pellets with 30% Addition of Cricket Powder [mg/100 g dw]
Gallic acid	5.10	0.60 ± 0.0220	0.605 ± 0.0124
Chlorogenic acid	6.40	17.31 ± 0.0761	17.33 ± 0.1178
Caffeic acid	6.90	1.12 ± 0.0320	1.127 ± 0.03411
Syringic acid	7.14	not detected	0.05 ± 0.0021
4-OH-benzoic acid	7.29	not detected	0.09 ± 0.0015
Vanillic acid	7.46	0.83 ± 0.0210	not detected
*p*-Coumaric acid	9.28	not detected	0.02 ± 0.0010
Sinapic acid	9.75	not detected	0.01→0.0005
Ferulic acid	9.90	0.264 ± 0.0119	0.304 ± 0.0147

**Table 3:** Results of extrusion cooking efficiency and energy consumption of snack pellets.

House Cricket Powder Addition [%]	Screw Rotation [RPM]	Moisture [%]	Q [kg/h]	SME [kWh/kg]
0	60	32	20.08 ± 0.37 ^a^	0.038 ± 0.001 ^e^
		36	21.84 ± 0.24 ^b^	0.034 ± 0.000 ^e^
	100	32	34.08 ± 1.25 ^d^	0.016 ± 0.001 ^ab^
		36	36.16 ± 0.28 ^e^	0.022 ± 0.000 ^bc^
10	60	32	20.64 ± 0.00 ^ab^	0.053 ± 0.000 ^f^
		36	20.48 ± 0.14 ^a^	0.048 ± 0.000 ^f^
	100	32	30.68 ± 0.25 ^c^	0.029 ± 0.000 ^cde^
		36	33.36 ± 0.24 ^d^	0.023 ± 0.000 ^bcd^
30	60	32	20.00 ± 0.28 ^a^	0.017 ± 0.000 ^ab^
		36	20.96 ± 0.50 ^ab^	0.031 ± 0.010 ^de^
	100	32	29.76 ± 0.24 ^c^	0.013 ± 0.001 ^ab^
		36	33.68 ± 0.14 ^d^	0.010 ± 0.006 ^a^

^a–f^—means indicated with similar letters in columns do not differ significantly at α = 0.05.

**Table 4:** Results for selected physical properties of snack pellets enriched with cricket powder processed in variable conditions (n = 3 ± SD).

House Cricket Powder Addition [%]	Screw Rotation [RPM]	Moisture [%]	WAI [g/g]	WSI [%]	Bulk Density [kg/m^3^]	Durability [%]
0	60	32	3.23 ± 0.00 ^abc^	8.24 ± 0.01 ^c^	420.70 ± 5.33 ^f^	98.14 ± 0.16 ^cdef^
		36	3.90 ± 0.01 ^abcde^	17.21 ± 0.00 ^d^	384.64 ± 4.40 ^e^	98.26 ± 0.42 ^def^
	100	32	3.98 ± 0.00 ^bcde^	7.54 ± 0.01 ^bc^	425.23 ± 13.31 ^f^	98.71 ± 0.43 ^f^
		36	4.07 ± 0.00 ^cde^	18.35 ± 0.00 ^d^	416.72 ± 5.40 ^f^	98.40 ± 0.34 ^ef^
10	60	32	4.53 ± 0.04 ^de^	3.81 ± 1.00 ^ab^	328.23 ± 15.12 ^cd^	97.41 ± 0.28 ^bcdef^
		36	3.03 ± 0.05 ^abc^	3.67 ± 2.29 ^ab^	310.07 ± 1.69 ^bc^	97.03 ± 0.58 ^abcdef^
	100	32	4.72 ± 0.12 ^e^	3.67 ± 0.91 ^ab^	394.93 ± 1.50 ^e^	96.43 ± 0.75 ^abcde^
		36	2.81 ± 0.14 ^ab^	3.76 ± 1.67 ^ab^	347.13 ± 2.47 ^d^	96.76 ± 0.45 ^abcdef^
30	60	32	3.31 ± 0.09 ^abc^	2.90 ± 1.33 ^a^	263.53 ± 1.40 ^a^	95.21 ± 0.27 ^a^
		36	3.52 ± 1.38 ^abcd^	6.14 ± 3.01 ^abc^	262.03 ± 1.81 ^a^	96.20 ± 0.51 ^abc^
	100	32	3.41 ± 0.08 ^abcd^	2.57 ± 1.79 ^a^	298.03 ± 1.00 ^b^	95.82 ± 0.22 ^ab^
		36	2.75 ± 0.08 ^a^	3.43 ± 0.38 ^ab^	266.67 ± 2.78 ^a^	96.42 ± 1.84 ^abcd^

^a–f^—means indicated with similar letters in columns do not differ significantly at α = 0.05.

**Table 5:** The proportional shares of individual components in the mixtures.

Raw Materials	Control Sample	10% Insect Powder	30% Insect Powder
Insect powder (%)	0	10	30
Potato starch (%)	82	72	52
Potato flakes (%)	15	15	15
Vegetable oil (%)	1	1	1
Sugar (%)	1	1	1
Salt (%)	1	1	1

**Table 6:** Temperature in different extruder-cooker sections.

House Cricket Powder Addition [%]	Screw Rotation [RPM]	Moisture [%]	Temperature of Section I [°C]	Temperature of Section II [°C]	Temperature of Section III [°C]	Temperature of Section IV [°C]	Extruder Die [°C]
0	60	32	44.90 ± 0.53	67.11 ± 0.35	66.09 ± 0.64	61.80 ± 0.41	62.60 ± 0.69
		36	47.99 ± 0.27	65.69 ± 0.32	66.50 ± 0.27	62.90 ± 0.11	64.51 ± 0.14
	100	32	45.11 ± 0.26	68.10 ± 0.67	67.21 ± 0.80	63.81 ± 0.34	63.20 ± 0.21
		36	49.49 ± 0.37	66.10 ± 0.10	66.80 ± 0.28	63.11 ± 0.16	62.60 ± 0.17
10	60	32	48.71 ± 0.19	65.70 ± 0.29	67.00 ± 0.17	64.61 ± 0.55	64.91 ± 0.13
		36	47.09 ± 0.28	64.80 ± 0.15	66.31 ± 0.27	64.40 ± 0.83	64.59 ± 0.66
	100	32	47.19 ± 0.11	68.00 ± 0.43	66.89 ± 0.33	64.91 ± 0.26	62.51 ± 0.28
		36	48.51 ± 0.11	65.49 ± 0.29	66.30 ± 0.20	64.20 ± 0.45	62.00 ± 0.19
30	60	32	45.80 ± 0.25	65.30 ± 0.69	66.29 ± 0.19	63.90 ± 0.42	64.89 ± 0.21
		36	46.00 ± 0.28	64.61 ± 0.18	66.20 ± 0.44	64.19 ± 0.43	64.80 ± 0.14
	100	32	44.90 ± 0.64	66.79 ± 0.58	66.30 ± 0.12	64.50 ± 0.13	61.90 ± 0.31
		36	47.39 ± 0.63	65.39 ± 0.61	65.91 ± 0.14	63.89 ± 0.15	62.50 ± 0.78

## Data Availability

The original contributions presented in the study are included in the article, and further inquiries can be directed to the corresponding author.
